# Responses of an Agricultural Soil Microbiome to Flooding with Seawater after Managed Coastal Realignment

**DOI:** 10.3390/microorganisms6010012

**Published:** 2018-01-26

**Authors:** Kamilla S. Sjøgaard, Thomas B. Valdemarsen, Alexander H. Treusch

**Affiliations:** 1 Department of Biology, University of Southern Denmark, 5230 Odense M, Denmark; ksjogaard@gmail.com (K.S.S.); valdemarsen@gmail.com (T.B.V.); 2 Nordcee, University of Southern Denmark, 5230 Odense M, Denmark

**Keywords:** Gyldensteen coastal lagoon, climate change, sea-level rise, nitrogen cycle, microbiome succession

## Abstract

Coastal areas have become more prone to flooding with seawater due to climate-change-induced sea-level rise and intensified storm surges. One way to cope with this issue is by “managed coastal realignment”, where low-lying coastal areas are no longer protected and instead flooded with seawater. How flooding with seawater impacts soil microbiomes and the biogeochemical cycling of elements is poorly understood. To address this, we conducted a microcosm experiment using soil cores collected at the nature restoration project site Gyldensteen Strand (Denmark), which were flooded with seawater and monitored over six months. Throughout the experiment, biogeochemical analyses, microbial community fingerprinting and the quantification of marker genes documented clear shifts in microbiome composition and activity. The flooding with seawater initially resulted in accelerated heterotrophic activity that entailed high ammonium production and net removal of nitrogen from the system, also demonstrated by a concurrent increase in the abundances of marker genes for ammonium oxidation and denitrification. Due to the depletion of labile soil organic matter, microbial activity decreased after approximately four months. The event of flooding caused the largest shifts in microbiome composition with the availability of labile organic matter subsequently being the most important driver for the succession in microbiome composition in soils flooded with seawater.

## 1. Introduction

Sea-level rise driven by climate change is expected to impact ~70% of the global coastlines during the 21st century [1]. The resulting increased amplitude and frequency of storm surges will lead to more incidences of broken coastal defenses and floods [2], and strategies to cope with this problem are required. Besides the classical strategy of improving coastal defenses, other ways of climate change adaption are under consideration [3,4,5]. An increasingly popular strategy is “managed coastal realignment”, where low-lying coastal areas are permanently flooded with seawater by the intentional breaching of existing dikes and building of new dikes further inland [6,7]. This generates buffer zones for storm surges, protecting the more valuable agricultural areas and settlements inland [8,9].

Soils are heterogeneous environments which are mainly oxic down to 75–100 cm depth [10,11,12] but also contain anoxic microniches [13]. Flooding with seawater will cause a shift towards anoxic conditions below a depth of few millimeters. This will limit soil organic matter (SOM) degradation to mainly anaerobic processes such as fermentation [14] and stimulate respiration with alternative electron acceptors, for instance sulfate (SO_4_^2^^−^) reduction [15,16] or denitrification [17,18]. Although many types of heterotrophic bacteria are facultative anaerobes and thus able to utilize alternative electron acceptors, the soil microbiome composition is expected to change drastically in response to the flooding. Seawater will increase the salinity in the soils as well as provide SO_4_^2^^−^ that can give SO_4_^2^^−^ reduction a competitive advantage over other anaerobic respiration pathways. Nevertheless, with the high abundance of nitrate (NO_3_^−^) in the flooded anoxic soils and the potential to perform full or partial denitrification present in many prokaryotes, it can also be expected that denitrification will become significant. The capability for denitrification is not confined to a specific group of Bacteria or Archaea [19], making predictions about this anaerobic respiration pathway based on taxonomic assignments using the 16S rRNA gene uncertain at best. Therefore, functional genes encoding enzymes in the denitrification pathway are used instead, with the genes for both the cytochrome *cd1*- (*nirS*) and the copper (*nirK*) nitrite (NO_2_^−^) reductase being molecular markers for the key step in denitrification, the reduction of NO_2_^−^ to nitric oxide [19].

The NO_3_^−^ used by the denitrifiers is often supplied by nitrifiers, which oxidize the ammonium (NH_4_^+^) released from organic nitrogen mineralization during biomass degradation. Classical bacterial nitrifiers oxidize NH_4_^+^ in two steps, with ammonia-oxidizing bacteria (AOB) comprised of species of the *Beta*- and *Gammaproteobacteria* catalyzing the first step from NH_4_^+^ to NO_2_^−^ [20]. *Nitrobacter* and *Nitrospira* species, collectively nitrite-oxidizing bacteria (NOB), generally carry out the second oxidation step from NO_2_^−^ to NO_3_^−^. Recently, bacterial species of the genus *Nitrospira* that can perform both steps of NH_4_^+^ oxidation, termed complete NH_4_^+^ oxidation (comammox), have been discovered in wastewater treatment plants [21,22]. *Nitrospira* capable of comammox seem to be widespread in many different ecosystems, including soils [23]. Besides Bacteria, Archaea of the phylum *Thaumarchaeota* can also nitrify, as revealed by metagenomics [24]. Depending on soil type and NH_4_^+^ concentration, these ammonia-oxidizing archaea (AOA) can outnumber AOB by a factor of up to 80 [25]. The commonly used functional marker gene for NH_4_^+^ oxidation of both Bacteria and Archaea is *amoA*, which encodes the ammonia monooxygenase subunit A.

Biogeochemical and microbial ecological studies on soils that are permanently flooded with seawater are extremely rare. Although wetlands and marshlands experiencing salinization are increasingly studied [26,27], microbial community dynamics in soil environments experiencing saltwater intrusions are still highly understudied. In the few studies available, no general trend of how the microbial communities respond was observed, instead the introduction of seawater or the increase in salinity seems to have triggered different responses in various systems. Shifts in microbial community composition were observed in agricultural soils that were flooded with seawater for two weeks and two months by a tsunami event [28]. *Acidobacteria* were most abundant in non-flooded soils (~35%), while in flooded soils *Proteobacteria* (30–67%) took over along with the appearance of sulfate oxidizing bacteria [28]. In wetland sediments experiencing salinization, the bacterial community showed only little responses while the methanogens (Archaea) changed significantly [29]. Freshwater lake sediment exposed to stepwise salinization, up to hypersalinity (90‰), over 60 days saw a significant shift in bacterial community composition with a succession of salt tolerant taxa [30]. To better understand the effects that the flooding with seawater has on soil ecosystems, more studies are needed, especially with a focus on rapid and permanently flooded soils and soils experiencing flooding with high frequency as result of sea level rise.

With the aim of studying the effects that permanent flooding with seawater has on soil microbiome composition and testing how SOM degradation is affected, we conducted a microcosm experiment in connection with a nature restoration project/managed coastal realignment at Gyldensteen Strand (Fyn, Denmark). Soil organic carbon (SOC) degradation was monitored as described by Sjøgaard et al. [31]. A peak of labile carbon degradation to carbon dioxide (CO_2_) occurred in the upper soil horizons within the first month of flooding. However, this degradation of SOC was insignificant compared to the available SOC pool, indicating the burial of the majority of terrestrial SOC and a minimal effect on atmospheric CO_2_ concentrations.

During the previous study [31] we also collected soil samples for molecular microbial ecological analyses as well as for biogeochemical analyses of the nitrogen mineralization dynamics. Here we present the observed changes of the bacterial community in response to the flooding as analyzed by terminal restriction fragment length polymorphism (T-RFLP) of the 16S rRNA gene. Further, the changes in the potential for nitrogen mineralization were assessed by quantifying the abundances of the bacterial and archaeal marker genes for NH_4_^+^ oxidation (*amoA*) and the two marker genes for NO_2_^−^ reduction (*nirK* and *nirS*). Together with rates of nitrogen mineralization, potential ammonia oxidation and denitrification, this dataset gives insight into the changes in nitrogen cycling in response to the flooding of soils with seawater.

## 2. Materials and Methods

### 2.1. Study Site

The study area at Gyldensteen (Fyn, Denmark) was a ~600 ha shallow intertidal habitat until 1871 when it was reclaimed for mainly agricultural use [32]. In March 2014, 211 ha of the area were permanently flooded with seawater by managed coastal realignment creating a shallow marine lagoon (for more details, see Sjøgaard et al. [31]). Sampling was conducted before the flooding at two stations in the lagoon with different land management histories. Station UC was an uncultivated reed swamp with moist soil and a high content of organic material. Station C had been cultivated, resulting in a homogeneous soil with only little organic material in the top layer.

### 2.2. Experimental Design and Sampling

The experimental design and sampling has been described previously [31]. In brief, 24 soil cores were collected in 30 cm long, 8 cm internal diameter stainless steel core liners from each station. All core liners were pushed 25 cm down in the soil, dug up and closed at both ends with rubber stoppers. In the laboratory, the headspaces of the soil cores were carefully flooded with seawater and transferred to 70 L incubation tanks also filled with 22–26‰ salinity seawater collected from the shoreline at Gyldensteen Strand at various time points during 2013–2014. During the whole experiment, the flooded cores were maintained at 15 °C and kept in darkness to avoid growth of phototrophs. The water in the tanks was rigorously aerated through air diffuser stones and 10–20 L of the seawater in the tanks were exchanged with fresh seawater every 14 days.

The experiment ran for six months with flux experiments (*n* = 13 over the six months) being conducted with three random soil cores from each station at various times (weekly in the first month, biweekly for the next three months and monthly in the last two months). Core sectioning was performed four times (one week, and two, four, and six months after the initial flooding) during the experiment. Three random soil cores from each station were sectioned at each time point. Soil from each core sectioning was used for biogeochemical- and molecular analyses as described below. Soil characteristics, contents of Fe(II) and Fe(III), rates of anoxic TCO_2_ (=CO_3_^2^^−^ + HCO_3_^−^ + H_2_CO_3_) and dissolved organic carbon (DOC) production, and SO_4_^2^^−^ reduction from this experiment have been described previously by Sjøgaard et al. [31] and are included in analyses here.

Additionally, seawater samples for molecular analyses were taken every time fresh seawater was collected at Gyldensteen Strand as well as from the incubation tank at every core sectioning time point. The water was filtered through 0.2 μm Supor^®^ 200 filters (Pall Corporation, New York, NY, USA) to collect biomass. Filters were stored at −20 °C until use.

### 2.3. Flux Experiments

Fluxes of NH_4_^+^ and NO_x_^−^ (=NO_3_^−^ + NO_2_^−^) between soil and overlying water were measured on three random cores from each station regularly during the experiment. The cores were each equipped with a Teflon-coated magnet, closed with a rubber stopper, placed around a central magnet rotating at 60 rpm and incubated for about 4 h in the dark. Water samples from the headspaces of the soil cores were taken at the beginning and at the end of incubations. Samples for analysis of nutrients (NH_4_^+^ and NO_x_^−^) were transferred to 20 mL vials and stored at −20 °C until analysis. NH_4_^+^ was measured by the salicylate-hypochlorite method [33]. NO_x_^−^ was measured with the method described by Grasshoff et al. [34] where NO_3_^−^ is reduced to NO_2_^–^ in a Cd column and the resulting NO_2_^–^, representing NO_x_^−^, was measured by the sulfanilamide reagent using a Lachat Quickchem 8500 Flow Injection Analyzer (Hach, Loveland, CO, USA). 

### 2.4. Core Sectioning

Core sectioning was performed by slicing each soil core into 6 depth intervals (0–1, 1–2, 2–5, 5–10, 10–15 and 15–20 cm). Soil from each depth interval was homogenized and triplicate soil samples of ~2 g were collected for molecular analysis. These subsamples were immediately frozen in liquid nitrogen and stored at −80 °C. Porewater was extracted from soil from each depth interval by centrifugation and GF/C filtration (Sigma-Aldrich, Darmstadt, Germany) in double centrifuge tubes at 500 g for 10 min. The porewater was stored at −20 °C until analysis for NH_4_^+^, NO_x_^−^ and Cl^−^. NH_4_^+^ and NO_x_^−^ were measured as described above. Cl^–^ was measured by ion chromatography on a Dionex ICS-2000 system (LabX, Midland, ON, Canada).

### 2.5. Anoxic Incubations (Jar Experiments)

Total microbial NH_4_^+^ production was measured in anoxic jar experiments [35,36]. After each core sectioning, remaining soil was pooled into four depth intervals (0–2, 2–5, 5–10 and 15–20 cm), homogenized and tightly packed into 6–8 scintillation vials (20 mL), which were closed with screw caps, buried and incubated in anoxic sediment at 15 °C to ensure an anoxic environment in the jars. In the following four weeks two jars were used every week for porewater extraction. The screw caps were changed to a perforated lid containing a GF/C filter and the jars were centrifuged upside-down in a centrifuge tube (10 min at 500 g). The extracted porewater was stored at −20 °C until analysis for NH_4_^+^ as described above.

### 2.6. Calculations

NH_4_^+^ production rates were calculated for the 0–2, 2–5, 5–10, 15–20 cm depths from the jar experiments by fitting the time-dependent concentration when the slopes of linear regressions were significant (*p* < 0.05). After correction for sediment porosity, the volume specific reaction rates in the individual depth layers (nmol cm^−3^ d^−1^) were calculated from the regression slopes. NH_4_^+^ production rates per area were calculated by depth integration over 0–20 cm and converted to area-specific units (mmol m^−2^ d^−1^). Linear data interpolation was used to estimate values for the depth interval 10–15 cm where rates were not measured. DIN (dissolved inorganic nitrogen = NH_4_^+^ + NO_x_^−^) fluxes were calculated as the sum of NH_4_^+^ and NO_x_^−^ fluxes. Potential denitrification rates were estimated as the difference between area-specific NH_4_^+^ production obtained in jar experiments and area-specific DIN efflux corrected for NH_4_^+^ accumulation in the porewater over time. In this calculation, we assumed the missing DIN was lost as gaseous nitrogen compounds (N_2_, N_2_O), which were not measured in the experiment. At station C, the DIN efflux was similar to total NH_4_^+^-production after four and six months indicating that denitrification was negligible. The potential NH_4_^+^ oxidation was estimated as the potential denitrification plus the area specific NO_x_^−^ efflux.

### 2.7. DNA Extraction

DNA was extracted from 0.3–0.4 g soil using the MoBio PowerLyzer^®^ PowerSoil^®^ DNA Isolation Kit (MoBio, Carlsbad, CA, USA) following the manufacturer’s instructions. DNA extractions of seawater samples were performed with the MoBio PowerWater^®^ DNA Isolation Kit following the manufacturer’s instructions. The DNA quality and concentration of the extracts were measured with a NanoDrop^®^ Spectrophotometer ND-1000 (NanoDrop Technologies, Wilmington, DE, USA). DNA was stored at −20 °C and the concentrations were adjusted to 5 ng/μL before further analyses.

### 2.8. Quantitative PCR

Abundances of Bacteria (16S rRNA), nitrifiers (bacterial and archaeal *amoA*) and denitrifiers (*nirK* and *nirS*) (Table S1) were estimated by quantitative polymerase chain reaction (qPCR). The qPCR was performed with RealQ Plus 2× Master Mix Green Without ROX^TM^ (Ampliqon, Odense, Denmark) in duplicate 25 μL reactions with 5–10 ng template and 25 pmol of each of the specific primers (Table S1). For thermal cycling a CFX Connect Real-Time System (BIO-RAD, Hercules, CA, USA) was used with the following PCR conditions: denaturation at 95 °C for 15 min followed by 40 cycles of denaturation at 95 °C for 20 s, annealing for 30 s with temperature depending on target gene (see Table S1), and extension at 72 °C for 30 s. A melt curve analysis completed the program, where the temperature was raised from 65 °C to 95 °C in 0.5 °C intervals at 5 s for each step. All reactions were run with standards of the corresponding target gene ranging from 10^1^ to 10^7^ copies per μL. The starting quantities of the genes were determined using the CFX Manager 3.0 software (BIO-RAD, Hercules, CA, USA) and were then standardized directly to copies g^−1^ dry soil.

### 2.9. T-RFLP Analysis

To get a general overview over the development of the bacterial community, 16S rRNA genes were analyzed by T-RFLP [37,38]. The genes were amplified using the fluorophore-labelled forward primer B27F FAM and the reverse primer U519R (Table S1) yielding a 530–550 bp fragment. The PCR was performed in 50 μL reactions with 10–20 ng template DNA, 20 pmol of each primer, 0.25 μL Taq DNA polymerase (5 U/μL, Thermo Scientific, Life Technologies, Carlsbad, CA, USA), 1.25 μL BSA (20 mg/mL, Thermo Scientific), 40 nmol deoxynucleotides and 25 mM MgCl_2_ in 10× *Taq* reaction buffer (Thermo Scientific). The PCR program consisted of an initial denaturing temperature of 98 °C for 2 min followed by 32 cycles of: denaturing at 94 °C for 20 s, annealing at 54 °C for 45 s, and extension at 72 °C for 45 s. A final extension step of 5 min at 72 °C completed the program. The PCR products were hydrolyzed with *Bsu*RI (10 U/μL, Thermo Scientific) using 30 μL PCR product in 70 μL reactions at 37 °C for 8 h, followed by a heat inactivation step of 80 °C for 20 min. The restriction fragments were purified with the GeneJET PCR Purification Kit (Thermo Scientific) and sent for capillary electrophoresis at the Uppsala Genome Center.

The sizes of the resulting T-RFs were determined with the Peak Scanner^®^ v1 software (Applied Biosystems, Foster City, CA, USA) using the internal size standard MapMarker 1000 (BioVentures, Murfreesboro, TN, USA). T-RFs within a size range of 50–550 bp were used for further analysis. Data were processed using T-RFLP analysis Expedited (T-REX) [39] by noise filtration at a factor of 1.15 based on peak area and remaining peaks were aligned with a clustering threshold of 0.6. A table of operational taxonomic units (OTUs) was created with fluorescence intensity (peak area) as a measure of abundance, which was standardized to relative abundance. Singleton OTUs and OTUs with a total abundance lower than 1% were filtered out, followed by log transformation of the relative abundances. Statistical analyses were performed in R [40] using the package vegan [41]. Bray Curtis dissimilarities were calculated for both samples and OTUs, analyzed by hierarchical clustering and nonmetric multidimensional scaling (NMDS) [42]. To test the significance of experimental factors (sampling station, depth and time point), the nonparametric analysis of variances using distance matrices (adonis) tests were performed. Furthermore, the explanatory power of the various environmental variables measured after the flooding with seawater was also evaluated by adonis tests.

## 3. Results

### 3.1. Soil Characteristics and Seawater Intrusion

The differences in land management at the two stations were clearly reflected in the soil characteristics. UC soil profiles showed steep depth gradients of water content, porosity and SOC, while soil profiles from station C were more homogenous and had three times lower SOC content than station UC. For further details regarding soil characteristics see Sjøgaard et al. [31].

**Figure 1:**
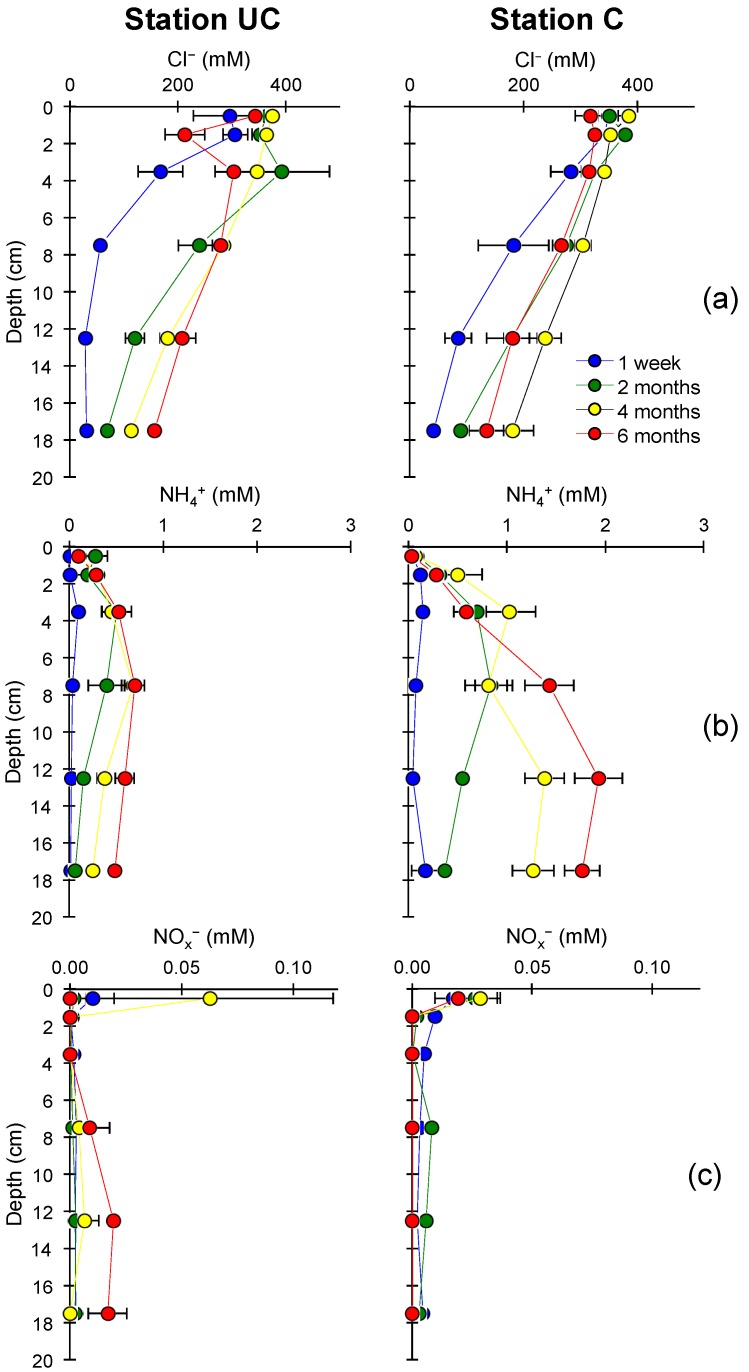
Depth profiles of: (**a**) chloride (Cl^−^); (**b**) ammonium (NH_4_^+^); and (**c**) nitrate + nitrite (NO_x_^−^) concentrations in porewater at each time point at the uncultivated (UC) and cultivated (C) stations. Error indicated as standard error of mean (SEM) (*n* = 3).

**Figure 2:**
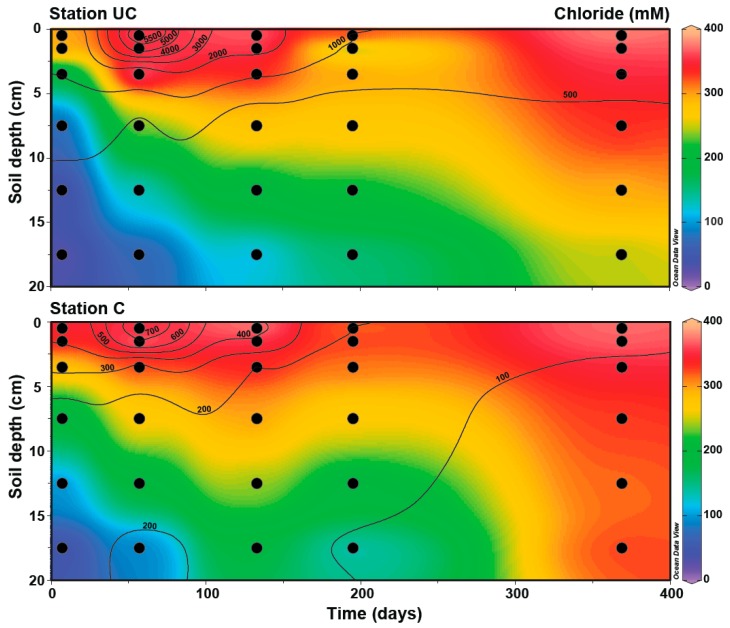
Rates of anoxic carbon dioxide production from Sjøgaard et al. [31] shown as contour lines (nmol cm^−3^ d^−1^) and chloride concentrations shown as a heat map for uncultivated (UC) and cultivated (C) stations at various soil depths at Week 1, and Months 2, 4, 6, and 12 after flooding with seawater. Each black dot represents a sampling point.

After flooding, a salinity gradient in the soil was observed from the porewater Cl^−^ concentrations (Figure 1a and Figure 2), with around 360 mM in the top layer decreasing to ~30 mM at 10–20 cm depth one week after flooding. Over time, the steepness of the Cl^−^ gradients decreased at both stations as seawater and associated solutes diffused down, with up to 157 ± 9 mM in UC soil after six months and 180 ± 38 mM in C soil after four months, at 15–20 cm depth. At both stations, full diffusion equilibrium in the 20 cm soil core was not reached during the experiment.

### 3.2. Nitrogen Mineralization

**Figure 3:**
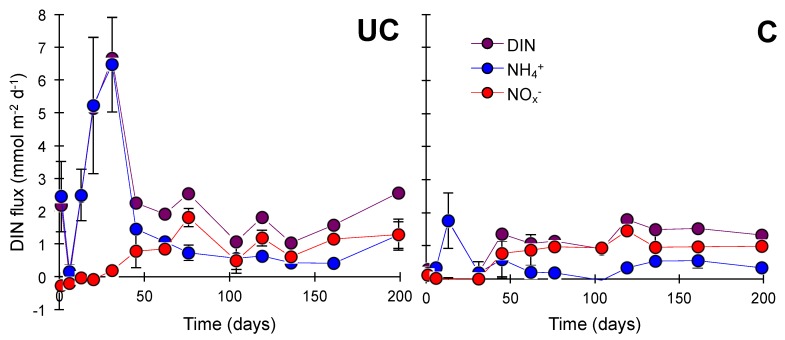
DIN effluxes (NH_4_^+^ efflux + NO_x_^−^ efflux) measured over 199 days on flooded cores from the uncultivated (UC) and cultivated (C) stations. Error bars indicate SEM (*n* = 3).

NH_4_^+^ effluxes were highest within the first 31 days after flooding, up to 6.5 ± 1.5 mmol m^−2^ d^−1^ and 1.8 ± 0.8 mmol m^−2^ d^−1^ at station UC and C, respectively, contributing to 97–100% and 58–100% of DIN fluxes (Figure 3) with remaining DIN consisting of NO_x_^−^. After 31 days NO_x_^−^ generally increased in importance and contributed 35–74% to DIN fluxes at station UC after 76 days and 57–100% at station C after 31 days. Simultaneously, the DIN effluxes stabilized at 0.9–2.6 mmol m^−2^ d^−1^ towards the end of the experiment at both stations.

Porewater concentrations of NH_4_^+^ (Figure 1b) showed similar time trends in both soils. NH_4_^+^ accumulated gradually from almost 0 mM at Week 1 to ~0.5 and ~1.6 mM at stations UC and C, respectively, with the highest concentrations being detected in the 5–15 cm depth horizon. NO_x_^−^ porewater concentrations were highest in the upper centimeter and were close to zero below.

**Figure 4:**
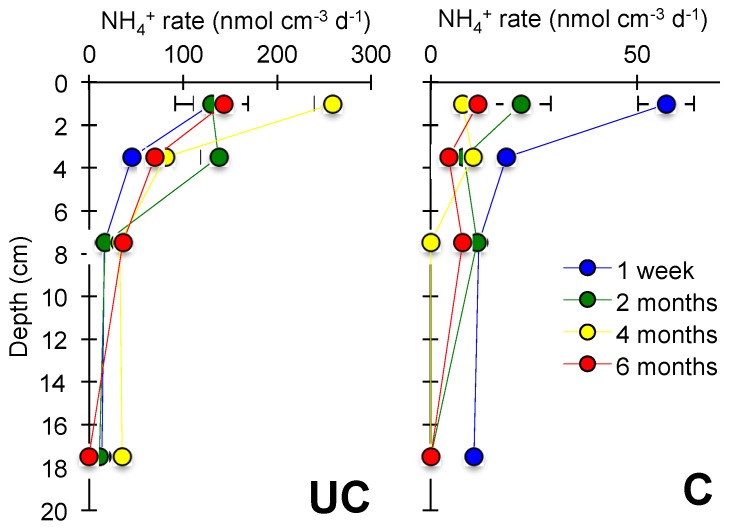
Volume specific ammonium (NH_4_^+^) production rates in the uncultivated (UC) and cultivated (C) soils at the four sampling time points. Note the different scales on the x-axes.

**Figure 5:**
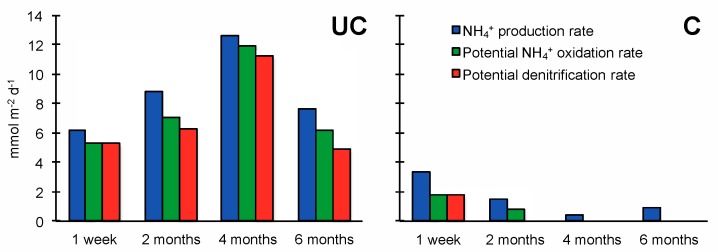
Area specific ammonium (NH_4_^+^) production rate, potential NH_4_^+^ oxidation and potential denitrification rates in uncultivated (UC) and cultivated (C) soil at the four sampling time points.

The anoxic jar experiments showed that the NH_4_^+^ production mainly occurred at 0–2 cm depth (Figure 4) and decreased with depth at both stations (Figure 5). At station UC, area specific NH_4_^+^ production rates (Figure 5) increased between Week 1 and Month 4 after flooding and were up to 12.7 mmol m^−2^ d^−1^. At C, the NH_4_^+^ production rate was highest in the beginning (3.4 mmol m^−2^ d^−1^) and decreased gradually over time and was 0.01 mmol m^−2^ d^−1^ by the end. Potential rates of denitrification and NH_4_^+^ oxidation suggested a great loss of nitrogen to the atmosphere ranging from 65–89% of the produced NH_4_^+^ at the station UC and around 53% at the station C. The potential rates showed similar temporal trends as the NH_4_^+^ production (Figure 5), while the potential NH_4_^+^ oxidation increased relative to potential denitrification over time.

### 3.3. Abundances of the Bacterial 16S rRNA, nirS, nirK and amoA Genes

**Figure 6:**
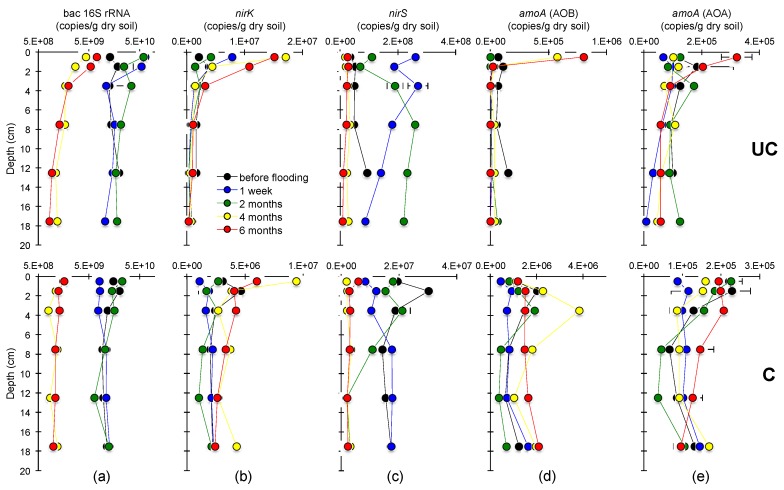
qPCR analysis illustrating abundance depth profiles of: (**a**) the bacterial 16S rRNA gene; (**b**) *nirK* gene; (**c**) *nirS* gene; (**d**) *amoA* gene of ammonia oxidizing bacteria (AOB); and (**e**) *amoA* gene of ammonia oxidizing archaea (AOA) at the uncultivated (UC) and cultivated (C) station from before flooding and the four sampling time points after flooding with seawater. The copy numbers were normalized directly to gram dry soil and bacterial 16S rRNA gene copy numbers were log transformed. Note different scales on the x-axes. Error indicated as standard error (*n* = 2).

The copy numbers of bacterial 16S rRNA genes, used as a proxy for bacterial abundance, ranged between 1.0 × 10^10^–6.4 × 10^10^ and 6.5 × 10^9^–2.3 × 10^10^ copies/g dry soil at station UC and C, respectively, which after four months had decreased to 8.2 × 10^8^–7.1 × 10^9^ and 7.8 × 10^8^–1.6 × 10^9^ copies/g dry soil (Figure 6a). The *nirS* gene showed the same trend over time as the bacterial 16S rRNA gene, but occurred at lower abundances, except at station UC where the *nirS* gene abundances were lower before flooding (Figure 6c). The *amoA* gene copy numbers at station C showed only a small increase over time. On the contrary, at station UC the bacterial *amoA* gene copy numbers increased significantly from zero to 5.7 × 10^5^ copies/g dry soil at 0–1 cm depth after four months, and archaeal *amoA* that increased from 6.7 × 10^4^ to 3.2 × 10^5^ copies/g dry soil after six months (Figure 6d,e). Furthermore, copy numbers of the *nirK* gene increased in similar fashion, reaching up to 1.7 × 10^7^ and 9.4 × 10^6^ copies/g dry soil at station UC and C, respectively, after four months (Figure 6b). 

### 3.4. Bacterial Community Composition (T-RFLP Analysis)

To monitor changes in the bacterial community composition over time, we analyzed the diversity of the 16S rRNA gene in the samples using T-RFLP. The estimated total richness amounted to 242 OTUs distributed over 70 samples, including the soil samples from UC, C and the seawater control samples. Analyzing the presence and absence of OTUs in these samples (Figure S1) showed that 111 OTUs were shared between the three groups, with an additional 60 OTUs shared only between UC and C. Only 7 and 13 OTUs were shared between the seawater samples, and UC and C, respectively.

**Figure 7:**
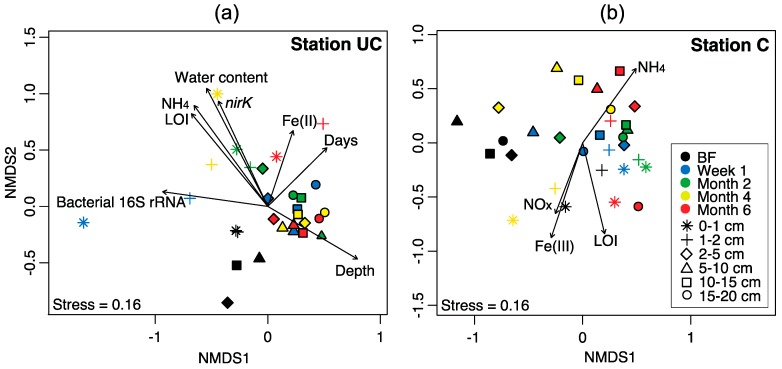
NMDS ordination of Bray–Curtis dissimilarities from T-RFLP analyses shown separately for: (**a**) station uncultivated (UC); and (**b**) cultivated (C). The samples are from before flooding (BF) and one week, two, four and six months after the soil cores were flooded with seawater. Colors correspond to the sampling time points and symbols represent the different soil depths. Environmental data were overlaid as vectors on the ordinations. Significantly correlating environmental data were soil depth in centimeter (depth), number of days after flooding (days), copy numbers of bacterial 16S rRNA, copy numbers of the *nirK* gene, percentage water content, ferrous iron content [Fe(II)], ferric iron content [Fe(III)], loss on ignition (LOI) a measure of soil organic matter content, ammonium content (NH_4_), nitrate+nitrite content (NO_x_).

Hierarchical clustering revealed overall differences between station UC and C (Figure S2). The NMDS ordinations (Figure 7) showed a general separation of samples from 0–2 cm depth and 2–20 cm depth, as well as clustering of samples from before flooding. At station UC, strong clustering was observed among all samples from before flooding, as well as for samples from 2–20 cm depth after flooding, exhibiting no differentiation considering time after flooding. On the other hand, samples from 0-2 cm depth did generally ordinate according to time after flooding at station UC (Figure 7). The NMDS ordination for station C had weaker clustering of samples, however still a general separation of samples from 0–2 and 2–20 cm was observed. Further, samples from before flooding at 2–20 cm depth and of samples from one week and two months after flooding showed weak clustering.

**Figure 8:**
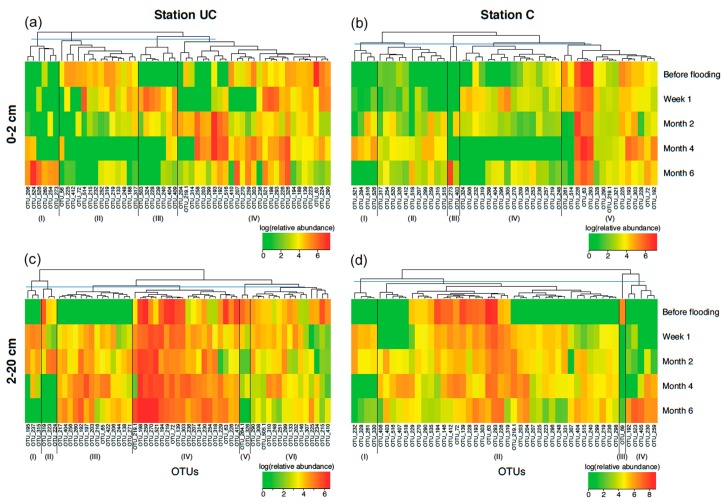
Heatmaps illustrating log transformed relative abundances of OTUs present at greater than 3% in: (**a**,**b**) soils at 0–2; and (**c**,**d**) 2–20 cm depth for the uncultivated (UC) and cultivated (C) stations at each sampling time point. Major modules of OTUs with similar abundance patterns were emphasized and numbered for each heatmap.

Soils from the 0–2 cm depth horizons at both stations showed higher variability in OTU composition over time (Figure 8a,b) relative to soils from 2–20 cm depth. The latter showed the highest change from before flooding to the time points after flooding (Figure 8c,d). Nearly none of the OTUs in UC soils from 0–2 cm depth persisted at high abundances from before flooding to Month 6 (Figure 8a). At station C, OTUs 63, 226 and 293 remained at high abundances throughout the experiment, though (Figure 8b). However, UC soil (0–2 cm) exhibited a rather clear gradual development in OTUs, occurring at different time points during the experiment from module II to III, then to the first part of IV and I. Similarly, at station C module IV was predominantly present around Week 1, while module I and II were mostly occurring around Month 4, but this development was less pronounced than in UC soil. Furthermore, module I in both soils at 2–20 cm depth occurred one week and two months after flooding, after which it disappeared (Figure 8c,d), while module IV at station C (2–20 cm) was only present at four/six months after flooding.

An adonis test (Table S2) indicated that most variability in OTU composition could be explained by the time points (*R*^2^ = 0.19, *p* = 0.001) rather than sampling station (*R*^2^ = 0.14, *p* = 0.001). When analyzing each station separately, the explanatory power of time points became even higher with *R*^2^ = 0.27 (*p* = 0.001) and *R*^2^ = 0.35 (*p* = 0.001) at station UC and C, respectively. Soil depth (0–2 and 2–20 cm) also had increased effect with *R*^2^ = 0.17 (*p* = 0.001) and *R*^2^ = 0.12% (*p* = 0.007) at station UC and C, respectively. Moreover, sampling time point in conjunction with depth showed some effect with *R*^2^ = 0.18 (*p* = 0.001) and *R*^2^ = 0.13 (*p* = 0.028) at station UC and C, respectively.

### 3.5. Correlation of Bacterial Community Structure with Environmental Parameters

Not much of the variability in the bacterial community composition observed from Week 1 to Month 6 after the soils were flooded with seawater could be explained by individual environmental parameters (note that the samples from before flooding had to be excluded due to missing environmental data). Instead, many small significant contributions were observed. The factor with the highest explanatory power of the variability was depth (UC: *R*^2^ = 0.23; C: *R*^2^ = 0.12; Table S3a), with time point of sampling explaining only little at either of the stations (UC: *R*^2^ = 0.06; C: *R*^2^ = 0.09). Other soil characteristics varying with depth such as water content (*R*^2^ = 0.07 at both stations) and Cl^−^ concentrations (UC: *R*^2^ = 0.05, C: not significant) explained surprisingly little of the community differences observed. Biogeochemical parameters reflecting microbial activity only partially explained the community compositions observed (Table S3b), with the highest values at station UC for the production rates of TCO_2_ (*R*^2^ = 0.20), DOC (*R*^2^ = 0.12) and NH_4_^+^ (*R*^2^ = 0.09) and at station C for the production rates of TCO_2_ (*R*^2^ = 0.10), DOC (*R*^2^ = 0.05), NH_4_^+^ (*R*^2^ = 0.05) and the SO_4_^2^^−^ reduction rates (*R*^2^ = 0.08) being the highest. Furthermore, the interactions between TCO_2_ and DOC production rates were also significant at both stations. Regarding how the qPCR data explain the bacterial community composition (Table S3c), the highest contributions came from the abundances of the bacterial 16S rRNA gene (UC: *R*^2^ = 0.17; C: *R*^2^ = 0.09). Furthermore, the *nirK* and *amoA* genes had significant explanatory power.

Environmental parameters showed significant correlations to the NMDS ordination of the T-RFLP dataset as seen in the overlaid vectors (Figure 7). The overlaid parameters that represent variation with time and depth were only significant at station UC. Environmental variables correlated with depth at station UC were water content, NH_4_^+^ concentration, LOI and abundance of the *nirK* gene. The abundance of bacterial 16S rRNA genes was positively correlated with surface samples and slightly to the samples from Week 1. Vectors representing the Fe(II) content pointed toward surface samples (Month 6), aligning with the number of days. Variation was highest at station C. Vectors for NO_x_^−^, Fe(III) and LOI generally pointed in the direction of samples from 0–2 cm depth, while NH_4_^+^ pointed in the opposite direction. These parameters roughly represent a distinction between soils from 0–2 and 2–20 cm depth.

## 4. Discussion

The flooding with seawater and its intrusion into the soil cores initiated some rapid changes in the soils, both physical and biogeochemical. Already one week after flooding, the soil was mostly anoxic and a salinity gradient was established with depth, as indicated by the Cl^−^ concentrations. Within one month after flooding, TCO_2_ and NH_4_^+^ effluxes peaked in response to highly accelerated microbial organic N mineralization activity. The long-term effects of the flooding were decreased microbial activity compared to immediately after flooding, and increased NH_4_^+^ oxidation relative to NH_4_^+^ production over time along with a concurrent shift in bacterial community composition.

### 4.1. Initially Accelerated Heterotrophic Activity and Its Later Decline

Station UC had accelerated carbon mineralization during the first month after flooding as described previously [31]. Several factors that changed after the flooding with seawater can be made responsible for this increase in microbial activity. Besides increased salinity, which has previously been suggested to stimulate microbial and enzymatic activity [43], the seawater provided high concentrations of SO_4_^2^^−^ in addition to the already high concentrations of reactive Fe(III) in the soil. Both are important electron acceptors in anaerobic carbon oxidation in anoxic marine environments [44]. The combination of high availability of degradable SOM and favorable conditions for anaerobic respiration resulted in an accelerated microbial activity within the first 30 days after flooding, a response that has been observed previously [15,45,46]. After the initial degradation of the highly labile SOC, the remaining SOC became more and more refractory over time since it mainly consisted of complex terrestrial plant material (e.g., lignocellulose), which is almost non-degradable under anoxic conditions.

### 4.2. Stimulated Nitrogen Cycling after Flooding with Seawater

Similar to the response in carbon cycling described above, the flooding also accelerated the microbial nitrogen cycling as reflected in the initially high NH_4_^+^ efflux at both stations. Later, the DIN effluxes shifted to a predominant contribution from NO_x_^−^, indicating that a microbial community of NH_4_^+^ oxidizers had proliferated after flooding in response to the high NH_4_^+^ levels, utilizing it for dissimilatory redox reactions. This was confirmed by an increase in the abundance of the *amoA* gene in the upper centimeter at station UC, especially at Month 4 and 6 after flooding. The abundance of *nirK* also increased, indicating a higher potential for denitrification. However, both AOBs and AOAs often contain both genes [24,47,48] and AOBs have been shown to perform coupled NH_4_^+^ oxidation–denitrification [49], which could explain the large nitrogen removal observed at this station.

The lower O_2_ requirements for microbial respiration in soil from station C probably caused a larger share of the produced NH_4_^+^ to be oxidized and hence lost as NO_x_^−^. This resulted in higher relative content of NO_x_^−^ in DIN-effluxes at station C (57–100%) than at station UC (33–74%). Interestingly, even though we observed increased NH_4_^+^ oxidation at station C, both archaeal and bacterial *amoA* gene abundances hardly changed over time. Considering that we observed a higher abundance of the *nirK* gene after four and six months without associated higher denitrification rate estimates, this indicates that at least some of the detected *nirK* genes originated from nitrifiers. While the primers used for the community profiling could not detect Archaea, the high effluxes of NO_x_^−^ relatively to NH_4_^+^ observed for station C could potentially be a result of AOAs. Although we cannot support this by an increasing copy number of the archaeal *amoA* gene over time because of potential primer biases, the higher relative efflux of NO_x_^−^ could be an indication for this. It has been suggested that AOAs are unable to perform coupled NH_4_^+^ oxidation and denitrification [50], which would explain the higher efflux.

We further observed that both the bacterial *nirS* and 16S rRNA genes decreased in copy numbers between Months 2 and 4. The abundance patterns of *nirS* were significant for the microbial communities at station UC (Table S3c). It is known that the capability to perform denitrification is ubiquitously distributed among Bacteria and Archaea with no recognizable correlation to the phylogenetic origin of the organisms [49]. As the same temporal trends in copy numbers were observed for both, the *nirS* and 16S rRNA genes, *nirS* possibly represented a wider spectrum of bacteria having a temporal community shift. The decreases in copy numbers for both 16S rRNA and *nirS* genes also illustrate a decrease in bacterial abundance in connection with the cessation of microbial activity. Furthermore, bacteria might carry the *nirS* gene in their genomes, without being active in denitrification that mainly takes place in the upper soil just below the oxic-anoxic interface where NO_x_^−^ is present [51,52].

A further indication that the microbial communities involved in nitrogen cycling are different at the two stations was revealed by the anoxic NH_4_^+^ production. Station UC showed increasing anoxic NH_4_^+^ production rates over time, reaching a maximum after four months, while at the station C the NH_4_^+^ production rates were highest initially and only decreased over time. This difference was likely due to higher SOM and thus heterotrophic activity in UC soil causing escalating NH_4_^+^ production [31]. This is in agreement with Santoro [53], who suggested increased nitrogen mineralization in interfaces between saltwater and freshwater. However, she also suggested decreased NH_4_^+^ oxidation and coupled NH_4_^+^ oxidation–denitrification [53], while we see a significantly increased potential for it. The total anoxic NH_4_^+^ production greatly exceeded the sum of DIN efflux and porewater accumulation at station UC, suggesting a substantial loss of fixed nitrogen to the atmosphere through coupled NH_4_^+^ oxidation–denitrification corresponding to 65–89% of the anoxic NH_4_^+^ production. Nitrogen could have been removed as N_2_, the end product of denitrification and anammox [54], but also as N_2_O, which is a potent greenhouse gas formed as a side product during NH_4_^+^ oxidation and denitrification [55,56,57]. Unfortunately, the gaseous nitrogen compound emissions were not monitored, leaving these for future experimental studies, for example using the nitrogen isotope pairing technique [58].

### 4.3. Composition of Microbiome Governed by Fluctuating Heterotrophic Activity

The event of flooding (difference from before flooding to one week after flooding) had the strongest effect on the composition of the microbiome. Previous studies similarly detected shifts in microbial community composition after introduction of seawater or increased salinity [28,59,60]. Asano et al. [28] studied bacterial communities in rice paddy soil one year after exposure to seawater due to the 2011 Tohoku Tsunami. They found that bacterial communities in soils not flooded with seawater clearly differed from those that were exposed to seawater, but soils flooded for two weeks and two months showed no significant differences. Similarly, in our study, the time after flooding became less significant, instead the most significant grouping factor was the depth (0–2 and 2–20 cm), alongside a significant relationship with the anoxic TCO_2_ and DOC production rates as a measure for microbial heterotrophic activity. This was especially clear for station UC that had the largest increase of microbial activity. Although some of the greatest changes occurred between before and after the soils were flooded, only very few OTUs disappeared completely. Microorganisms can be unable to cope with change in osmotic pressure [61] and/or be obligate aerobes, and these would probably have died off due to increased salinity and anoxic conditions introduced to the soil environment. Furthermore, because salinity is among the most important factors in determining the global distributions of microbial communities [62,63], we expected to observe changes in the microbiome that could be directly correlated to Cl^−^ concentration. However, only a minimal to no effect was detected, suggesting that salinity is only one of many factors shaping the microbial community in our experiment. The persistence of many OTUs after flooding could have multiple reasons. There is a good chance that some microbes entered dormancy, which is a common stress response caused by changes in environmental conditions [64], and hence were still detectable in the community. Further, newly introduced species and species previously present might have the same terminal fragment length, making them indistinguishable with the T-RFLP method we used [38]. The observed increase in the diversity of OTUs was most likely caused by marine bacteria introduced with the seawater intrusion, however, it is also possible that bacteria already present in the soil at very low abundances before flooding were triggered to proliferate by the event.

We also observed a significant relationship between the variability in community composition and bacterial 16S rRNA gene abundances showing increased cell numbers after flooding (Week 1 and Month 2), thus suggesting initial bacterial growth followed by a crash. Together with the accelerated heterotrophic activity, this indicates dynamics of opportunistic heterotrophic communities and so-called *boom and bust* populations that are likely shown by the modules in Figure 8: (a) III; (b) IV; (c) I; and (d) I. The OTUs of these modules almost exclusively occurred at Week 1 and Month 2 after flooding and were potentially opportunists. Generally, they were different, both among stations and depth horizons, suggesting that the various environments here stimulated different communities. For instance, the surface soils (0–2 cm) with their higher content of SOM seem to accelerate heterotrophic microbial activity, but are also more susceptible to changes in the environment due to immediate contact with the overlaying water as well as the steep redox gradients present. With the changes in the microbial community structure, most likely also the activity of extracellular enzymes involved in decomposition of SOM increased in parallel to the salinity, an effect that Morrissey et al. [43] also observed in tidal wetlands.

Altogether this suggests that newly introduced or low abundant bacterial species proliferated rather quickly in the soil, exhibiting opportunistic behavior reflecting the accelerated heterotrophic activity observed, which was more pronounced for station UC, both in the community composition and the carbon mineralization rates.

### 4.4. Implications and Future Studies

In our microcosms, we observed a major DIN release to the overlying water during the first 30 days of flooding, which in real world scenarios might cause eutrophication events. Indeed, throughout the first summer after flooding, massive algae blooms were observed in the lagoon at Gyldensteen Strand [65], which only at times of strong winds were flushed out beyond the shore. In the second summer, no indications of a similar event were observed [65], thus a release/leaching of soil nutrients must have occurred as a short-term response (30 days), while remaining nutrients in SOM are retained due to decreased microbial activity [31] and perhaps buffered within the system. Furthermore, the large anoxic NH_4_^+^ production and relatively minimal DIN efflux observed two and four months after flooding (calculated potential denitrification) indicated latent DIN that could have been released. However, it was heavily suppressed by denitrification and thereby a loss of fixed nitrogen (in this case DIN), mitigating eutrophication.

Another important aspect of nature restoration projects like the one at Gyldensteen Strand is the potential emission of greenhouse gases. While we observed in our experiment an initial strong microbial activity with potential for the production of greenhouse gases such as CO_2_, CH_4_ and N_2_O [45,66], this activity quickly ceased. However, the indications for extremely high rates of NH_4_^+^ oxidation and denitrification at station UC could suggest a high production of the side product of these pathways, N_2_O, potentially jeopardizing the effect that the flooding of soils has on CO_2_ emissions. Therefore, future studies need to elucidate the role of the nitrifier and denitrifier communities and their activity in more detail in order to get a holistic picture of the impact that flooding of soils with seawater has on the biogeochemical cycling of elements and the release of greenhouse gases.

## Supplementary Materials

The following are available online at http://www.mdpi.com/2076-2607/6/1/12/s1.

Supplementary File 1

## References

[1] Church J.A., Clark P.U., Cazenave A., Gregory J.M., Jevrejeva S., Levermann A., Merrifield M.A., Milne G.A., Nerem R.S., Nunn P.D., Stocker T.F., Qin D., Plattner G.-K., Tignor M., Allen S.K., Boschung J.A., Nauels Y. (2013). Sea level change. Climate Change: The Physical Science Basis. Contribution of Working Group I to the Fifth Assessment Report of the Intergovernmental Panel on Climate Change.

[2] FitzGerald D.M., Fenster M.S., Argow B.A., Buynevich I.V. (2008). Coastal impacts due to sea-level rise. Annu. Rev. Earth Planet. Sci..

[3] Hinkel J., Lincke D., Vafeidi A., Perrette M., Nicholls R., Tol R., Marzeion B., Fettweis X., Ionescu C., Levermann A. (2014). Coastal flood damage and adaptation costs under 21st century sea-level rise. Proc. Natl. Acad. Sci. USA.

[4] Lichter M., Vafeidis A.T., Nicholls R.J., Kaiser G. (2011). Exploring data-related uncertainties in analyses of land area and population in the “low-elevation coastal zone” (LECZ). J. Coast. Res..

[5] Nicholls R., Hanson S.E., Lowe J.A., Warrick R.S., Lu X., Long A.J. (2014). Sea-level scenarios for evaluating coastal impacts. Wiley Interdiscip. Rev. Clim. Chang..

[6] Cooper N.J. (2003). The use of ‘managed retreat’ in coastal engineering. Proc. Inst. Civ. Eng..

[7] French J.R. (2008). Hydrodynamic modelling of estuarine flood defence realignment as an adaptive management response to sea-level rise. J. Coast. Res..

[8] Wolters M., Garbutt A., Bakker J.P. (2005). Salt-marsh restoration: Evaluating the success of de-embankments in North-west Europe. Biol. Conserv..

[9] Pethick J. (2002). Estuarine and tidal wetland restoration in the United Kingdom: Policy vs. practice. Restor. Ecol..

[10] Dziejowski J.E., Rimmer A., Steenhuis T.S. (1997). Preferential movement of oxygen in soils?. Soil Sci. Soc. Am. J..

[11] MacDonald J.D., Costello L.R., Berger T. (1993). An evaluation of soil aeration status around healthy and declining oaks in an urban environment in California. J. Aboriculture.

[12] Neira J., Ortiz M., Morales L., Acevedo E. (2015). Oxygen diffusion in soils: Understanding the factors and processes needed for modeling. Chil. J. Agric. Res..

[13] Brussaard L., Behan-Pelletier V.M., Bignell D.E., Brown V.K., Didden W., Folgarait P., Fragoso C., Freckman D.W., Gupta V.V.S.R., Hattori T. (1997). Biodiversity and ecosystem functioning in soil. Ambio.

[14] Valdemarsen T., Kristensen E. (2010). Degradation of dissolved organic monomers and short-chain fatty acids in sandy marine sediment by fermentation and sulfate reduction. Geochim. Cosmochim. Acta.

[15] Weston N.B., Dixon R.E., Joye S.B. (2006). Ramifications of increased salinity in tidal freshwater sediments: Geochemistry and microbial pathways of organic matter mineralization. J. Geophys. Res. Biogeosci..

[16] Sutton-Grier A.E., Keller J.K., Koch R., Gilmour C., Megonigal J.P. (2011). Electron donors and acceptors influence anaerobic soil organic matter mineralization in tidal marshes. Soil Biol. Biochem..

[17] Arnosti C. (2011). Microbial extracellular enzymes and the marine carbon cycle. Ann. Rev. Mar. Sci..

[18] Glud R.N. (2008). Oxygen dynamics of marine sediments. Mar. Biol. Res..

[19] Philippot L. (2002). Denitrifying genes in bacterial and archaeal genomes. Biochim. Biophys. Acta.

[20] Purkhold U., Pommerening-Roser A., Juretschko S., Schmid M.C., Koops H.P., Wagner M. (2000). Phylogeny of all recognized species of ammonia oxidizers based on comparative 16S rRNA and *amoA* sequence analysis: Implications for molecular diversity surveys. Appl. Environ. Microbiol..

[21] Daims H., Lebedeva E.V., Pjevac P., Han P., Herbold C., Albertsen M., Jehmlich N., Palatinszky M., Vierheilig J., Bulaev A. (2015). Complete nitrification by *Nitrospira* bacteria. Nature.

[22] Van Kessel M.A., Speth D.R., Albertsen M., Nielsen P.H., Op den Camp H.J., Kartal B., Jetten M.S., Lucker S. (2015). Complete nitrification by a single microorganism. Nature.

[23] Pjevac P., Schauberger C., Poghosyan L., Herbold C.W., van Kessel M., Daebeler A., Steinberger M., Jetten M.S.M., Lucker S., Wagner M. (2017). *amoA*-targeted polymerase chain reaction primers for the specific detection and quantification of comammox *Nitrospira* in the environment. Front. Microbiol..

[24] Treusch A.H., Leininger S., Kletzin A., Schuster S.C., Klenk H.P., Schleper C. (2005). Novel genes for nitrite reductase and amo-related proteins indicate a role of uncultivated mesophilic crenarchaeota in nitrogen cycling. Environ. Microbiol..

[25] Verhamme D.T., Prosser J.I., Nicol G.W. (2011). Ammonia concentration determines differential growth of ammonia-oxidising archaea and bacteria in soil microcosms. ISME J..

[26] Herbert E.R., Boon P., Burgin A.J., Neubauer S.C., Franklin R.B., Ardón M., Hopfensperger K.N., Lamers L.P.M., Gell P. (2015). A global perspective on wetland salinization: Ecological consequences of a growing threat to freshwater wetlands. Ecosphere.

[27] Roman C.T., Burdick D.M. (2012). Tidal Marsh Restoration: A Synthesis of Science and Management.

[28] Asano R., Nakai Y., Kawada W., Shimura Y., Inamoto T., Fukushima J. (2013). Seawater inundation from the 2011 Tohoku tsunami continues to strongly affect soil bacterial communities 1 year later. Microb. Ecol..

[29] Baldwin D.S., Rees G.N., Mitchell A.M., Watson G., Williams J. (2006). The short-term effects of salinization on anaerobic nutrient cycling and microbial community structure in sediment from a freshwater wetland. Wetlands.

[30] Zhang L., Gao G., Tang X., Shao K. (2014). Can the freshwater bacterial communities shift to the “marine-like” taxa?. J. Basic Microbiol..

[31] Sjøgaard K.S., Treusch A.H., Valdemarsen T.B. (2017). Carbon degradation in agricultural soils flooded with seawater after managed coastal realignment. Biogeosciences.

[32] Stenak M. (2005). Inddæmningerne på Nordfyn. De Inddæmmede Landskaber—En Historisk Geografi.

[33] Bower C., Holm-Hansen T. (1980). A salicylate-hypochlorite method for determining ammonia in seawater. Can. J. Fish Aquat. Sci..

[34] Grasshoff K., Kremling K., Ehrhardt M. (1999). Methods of Seawater Analysis.

[35] Kristensen E., Hansen K. (1995). Decay of plant detritus in organic-poor marine sediment: Production rates and stoichiometry of dissolved C and N compounds. J. Mar. Res..

[36] Quintana C.O., Kristensen E., Valdemarsen T. (2013). Impact of the invasive polychaete *Marenzelleria viridis* on the biogeochemistry of sandy marine sediments. Biogeochemistry.

[37] Liu W.T., Marsh T.L., Cheng H., Forney L.J. (1997). Characterization of microbial diversity by determining terminal restriction fragment length polymorphisms of genes encoding 16S rRNA. Appl. Environ. Microbiol..

[38] Nocker A., Burr M., Camper A.K. (2007). Genotypic microbial community profiling: A critical technical review. Microb. Ecol..

[39] Culman S.W., Bukowski R., Gauch H.G., Cadillo-Quiroz H., Buckley D.H. (2009). T-rex: Software for the processing and analysis of t-rflp data. BMC Bioinform..

[40] R-Core-Team (2016). R: A Language and Environment for Statistical Computing.

[41] Oksanen J., Blanchet F.G., Friendly M., Kindt R., Legendre P., McGlinn D., Minchin P.R., O’Hara R.B., Simpson G.L., Solymos P. (2017). Vegan: Community Ecology Package. https://cran.R-project.Org/package=vegan.

[42] Rees G.N., Baldwin D.S., Watson G.O., Perryman S., Nielsen D.L. (2004). Ordination and significance testing of microbial community composition derived from terminal restriction fragment length polymorphisms: Application of multivariate statistics. Antonie Leeuwenhoek.

[43] Morrissey E.M., Gillespie J.L., Morina J.C., Franklin R.B. (2014). Salinity affects microbial activity and soil organic matter content in tidal wetlands. Glob. Chang. Biol..

[44] Canfield D.E., Jorgensen B.B., Fossing H., Glud R., Gundersen J., Ramsing N.B., Thamdrup B., Hansen J.W., Nielsen L.P., Hall P.O. (1993). Pathways of organic carbon oxidation in three continental margin sediments. Mar. Geol..

[45] Weston N.B., Vile M.A., Neubauer S.C., Velinsky D.J. (2011). Accelerated microbial organic matter mineralization following salt-water intrusion into tidal freshwater marsh soils. Biogeochemistry.

[46] Neubauer S.C., Franklin R.B., Berrier D.J. (2013). Saltwater intrusion into tidal freshwater marshes alters the biogeochemical processing of organic carbon. Biogeosciences.

[47] Cantera J.J., Stein L.Y. (2007). Molecular diversity of nitrite reductase genes (*nirK*) in nitrifying bacteria. Environ. Microbiol..

[48] Bartossek R., Nicol G.W., Lanzen A., Klenk H.P., Schleper C. (2010). Homologues of nitrite reductases in ammonia-oxidizing archaea: Diversity and genomic context. Environ. Microbiol..

[49] Zumft W.G. (1997). Cell biology and molecular basis of denitrification. Microbiol. Mol. Biol. Rev..

[50] Stieglmeier M., Mooshammer M., Kitzler B., Wanek W., Zechmeister-Boltenstern S., Richter A., Schleper C. (2014). Aerobic nitrous oxide production through *N*-nitrosating hybrid formation in ammonia-oxidizing archaea. ISME J..

[51] Middelburg J.J., Soetaert K., Herman P.M.J., Heip C.H.R. (1996). Denitrification in marine sediments: A model study. Glob. Biogeochem. Cycles.

[52] Seitzinger S.P. (1988). Denitrification in freshwater and coastal marine ecosystems: Ecological and geochemical significance. Limnol. Oceanogr..

[53] Santoro A.E. (2010). Microbial nitrogen cycling at the saltwater-freshwater interface. Hydrogeol. J..

[54] Thamdrup B., Dalsgaard T. (2002). Production of N_2_ through anaerobic ammonium oxidation coupled to nitrate reduction in marine sediments. Appl. Environ. Microbiol..

[55] Thamdrup B. (2012). New pathways and processes in the global nitrogen cycle. Annu. Rev. Ecol. Evol. Syst..

[56] Kampschreur M.J., Temmink H., Kleerebezem R., Jetten M.S., van Loosdrecht M.C. (2009). Nitrous oxide emission during wastewater treatment. Water Res..

[57] Guo J., Peng Y., Wang S., Ma B., Ge S., Wang Z., Huang H., Zhang J., Zhang L. (2013). Pathways and organisms involved in ammonia oxidation and nitrous oxide emission. Crit. Rev. Environ. Sci. Technol..

[58] Nielsen L.P. (1992). Denitrification in sediment determined from nitrogen isotope pairing. FEMS Microbiol. Ecol..

[59] Berga M., Szekely A.J., Langenheder S. (2012). Effects of disturbance intensity and frequency on bacterial community composition and function. PLoS ONE.

[60] Langenheder S., Ragnarsson H. (2007). The role of environmental and spatial factors for the composition of aquatic bacterial communities. Ecology.

[61] Csonka L.N. (1989). Physiological and genetic responses of bacteria to osmotic stress. Microbiol. Rev..

[62] Auguet J.C., Barberan A., Casamayor E.O. (2009). Global ecological patterns in uncultured archaea. ISME J..

[63] Lozupone C.A., Knight R. (2007). Global patterns in bacterial diversity. Proc. Natl. Acad. Sci. USA.

[64] Lennon J.T., Jones S.E. (2011). Microbial seed banks: The ecological and evolutionary implications of dormancy. Nat. Rev. Microbiol..

[65] Kristensen E., Flindt M.R., Thorsen S.W., Holmer M., Valdemarsen T. (2016). Gyldensteen strand—Fra agerland til kystlagune. Vand Jord.

[66] Conrad R. (1996). Soil microorganisms as controllers of atmospheric trace gases (H_2_, CO, CH_4_, OCS, N_2_O, and NO). Microbiol. Rev..

