# Supplementary material for: Responses of an Agricultural Soil Microbiome to Flooding with Seawater after Managed Coastal Realignment
Source: Microorganisms. 2018 Jan 26;6(1):12. doi: 10.3390/microorganisms6010012 (PMC5874626; doi:10.3390/microorganisms6010012)
Supplement: Supplementary File 1 [file microorganisms-06-00012-s001.pdf]

**Table S1** Description of primers used for T-RFLP and qPCR analysis.

| Target organism | Gene | Primer | Sequence (5'-3') | Annealing temperature (°C) | Reference |
|---|---|---|---|---|---|
| Bacteria | 16S rRNA | B27F | AGR GTT YGA TYM TGG CTC AG | 54 | Mason et al (2009) |
| | | U519R | TTA CCG CGG CKG CTG | | Ovreås et al (1997) |
| Bacterial nitrifiers | *amoA* | AOB-amoA-1F | GGG GTT TCT ACT GGT GGT | 60 | Rotthauwe et al (1997) |
| | | AOB-amoA-2R | CCC CTC KGS AAA GCC TTC TTC | | |
| Archaeal nitrifiers | *amoA* | AOA-amoA-f | CTG AYT GGG CYT GGA CAT C | 59 | Coolen et al (2007) |
| | | AOA-amoA-r | TTC TTC TTT GTT GCC CAG TA | | |
| Denitrifiers | *nirK* | F1aCu | ATC ATG GTS CTG CCG CG | 58.3 | Throbäck et al (2004) |
| | | R3Cu | GCC TCG ATC AGR TTG TGG TT | | |
| Denitrifiers | *nirS* | cd3aF | GTS AAC GTS AAG GAR ACS GG | 58.3 | Throbäck et al (2004) |
| | | R3cd | GAS TTC GGR TGS GTC TTG A | | |

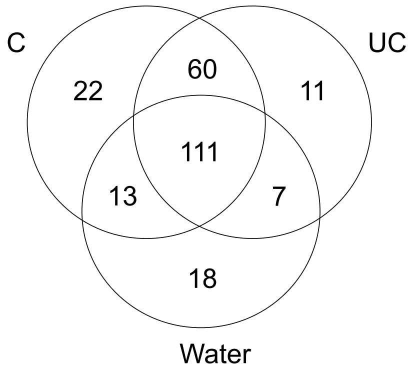


**Figure S1** Venn-diagram showing OTU distribution over all samples (n = 70), including water samples from Gyldensteen, which the cores were flooded with, water samples of the tank at each core sectioning time point as well as the soil samples from before and after flooding. Total number of OTUs was 242.

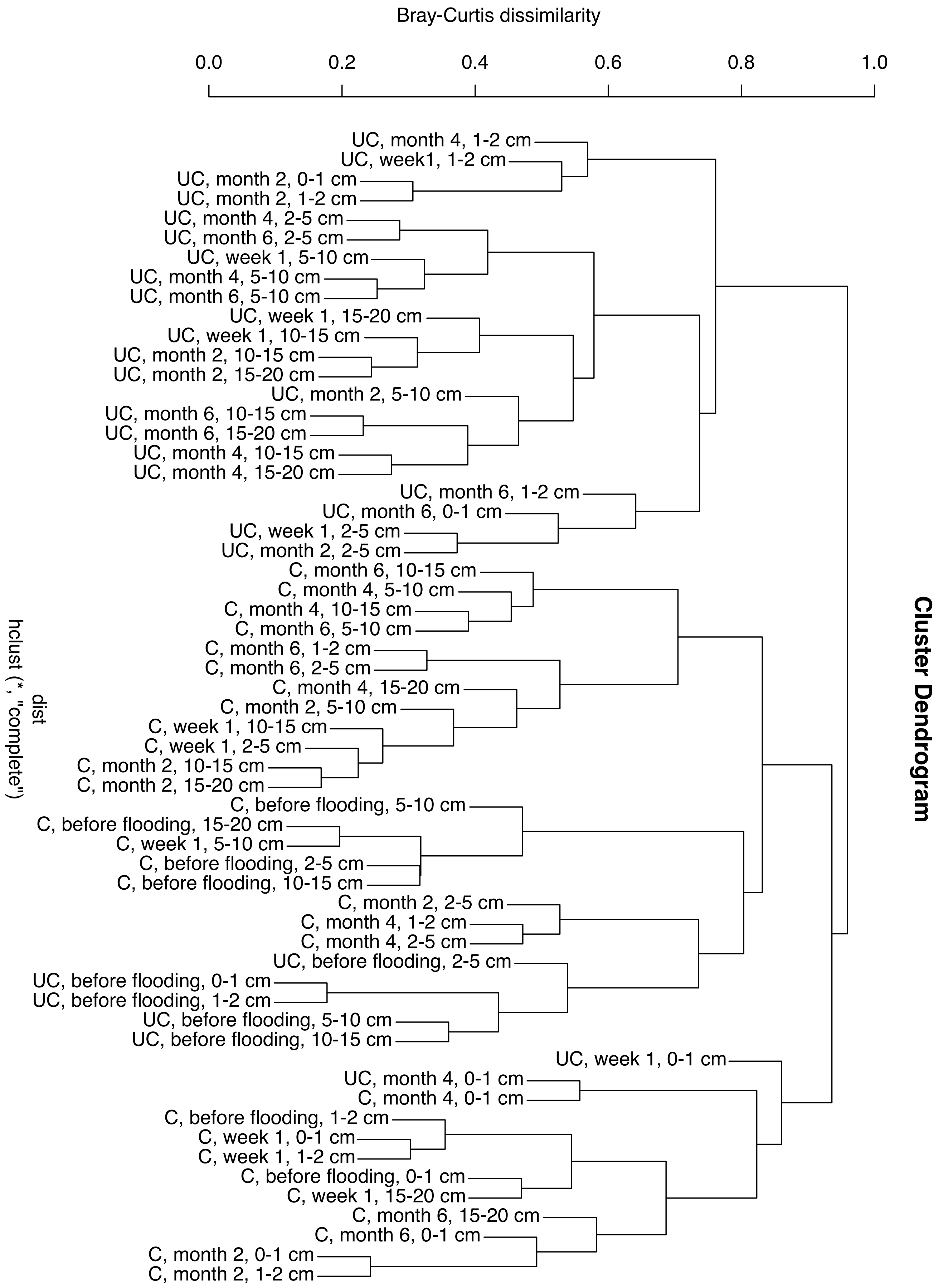


**Figure S2** Hierarchical clustering dendrogram of all soil samples.

**Table S2** Adonis test results of experimental grouping factors: Time point (before flooding, week one, month two, four and six), station (uncultivated [UC] and cultivated [C]), depth categories (0-2 and 2-20 cm depth). Factors where tested as (**a**) crossed for the stations together and (**b**) separately.

(a)

| Test variable | $R^2$ | p-value |
|---|---|---|
| Time point | 0.19 | 0.001 |
| Station | 0.14 | 0.001 |
| Depth | 0.08 | 0.001 |
| Time point : Station | 0.08 | 0.001 |
| Time point : Depth | 0.09 | 0.001 |
| Station : Depth | 0.04 | 0.001 |
| Time point : Station : Depth | 0.05 | ns |

(b)

| Test variable | Station UC | | Station C | |
|---|---|---|---|---|
| | $R^2$ | p-value | $R^2$ | p-value |
| Time point | 0.28 | 0.001 | 0.34 | 0.001 |
| Depth | 0.17 | 0.001 | 0.12 | 0.001 |
| Time point : Depth | 0.18 | 0.001 | 0.13 | 0.028 |

**Table S3** Adonis test results of environmental variables for time points after flooding. (**a**) The experimental factors depth (0-2 and 2-20 cm depth), time point (week one, month two, four and six) and soil characteristics were tested together. (**b**) Significance of total carbon dioxide ($TCO_2$), dissolved organic carbon (DOC), ammonium ($NH_4^+$) production rates, and reduction rates of sulfate ($SO_4^{2-}$) were tested. Furthermore, (**c**) abundances of the bacterial 16S rRNA, *nirK*, *nirS*, bacterial and archaeal *amoA* genes were tested. Factors and variables were tested as crossed for the uncultivated (UC) and cultivated (C) stations separately. Only crossed factors that were significant are shown. Not significant $R^2$ values are indicated by p-value = ns.

| (a) Test variable | Station UC | | Station C | |
|---|---|---|---|---|
| | $R^2$ | p-value | $R^2$ | p-value |
| Depth | 0.23 | 0.001 | 0.12 | 0.005 |
| Time point | 0.06 | 0.005 | 0.09 | 0.006 |
| Water content (%) | 0.07 | 0.005 | 0.07 | 0.025 |
| Chloride (mM) | 0.05 | 0.010 | 0.05 | ns |
| Depth : Time point | 0.07 | 0.004 | 0.02 | ns |
| Depth : Water content (%) | 0.05 | 0.023 | 0.06 | ns |
| Depth : Chloride (mM) | 0.08 | 0.001 | 0.03 | ns |
| Depth : Time point : Water content (%) | 0.06 | 0.007 | 0.04 | ns |
| Depth : Water content (%) : Chloride (mM) | 0.05 | 0.015 | 0.05 | ns |

| (b) Test variable | Station UC | | Station C | |
|---|---|---|---|---|
| | $R^2$ | p-value | $R^2$ | p-value |
| Anoxic $TCO_2$ production rate | 0.20 | 0.001 | 0.10 | 0.001 |
| Anoxic DOC production rate | 0.12 | 0.002 | 0.05 | 0.026 |
| Anoxic $NH_4^+$ production rate | 0.09 | 0.003 | 0.05 | 0.013 |
| Anoxic $SO_4^{2-}$ reduction rate | 0.03 | ns | 0.08 | 0.001 |
| Anoxic $TCO_2$ production rate : Anoxic DOC production rate | 0.08 | 0.003 | 0.12 | 0.001 |
| Anoxic $TCO_2$ production rate : Anoxic $NH_4^+$ production rate | 0.04 | ns | 0.04 | 0.037 |
| Anoxic DOC production rate : Anoxic $NH_4^+$ production rate | 0.02 | ns | 0.05 | 0.011 |
| Anoxic $TCO_2$ production rate : Anoxic $SO_4^{2-}$ reduction rate | 0.02 | ns | 0.06 | 0.004 |
| Anoxic DOC production rate : Anoxic $SO_4^{2-}$ reduction rate | 0.04 | ns | 0.06 | 0.006 |

| (c) Test variable | Station UC | | Station C | |
|---|---|---|---|---|
| | $R^2$ | p-value | $R^2$ | p-value |
| Bacterial 16S rRNA gene | 0.17 | 0.001 | 0.09 | 0.032 |
| *nirK* gene | 0.16 | 0.001 | 0.11 | 0.002 |
| *nirS* gene | 0.08 | 0.005 | 0.05 | ns |
| *amoA* gene (AOB) | 0.11 | 0.001 | 0.10 | 0.004 |
| *amoA* gene (AOA) | 0.05 | ns | 0.09 | 0.012 |
| *nirK* gene : *nirS* gene | 0.08 | 0.008 | 0.02 | ns |
| *nirK* gene : *nirS* gene : *amoA* gene (AOA) | 0.06 | 0.039 | 0.03 | ns |
